# *ARumenamides*: A novel class of potential antiarrhythmic compounds

**DOI:** 10.3389/fphar.2022.976903

**Published:** 2022-09-28

**Authors:** Mena Abdelsayed, Dana Page, Peter C. Ruben

**Affiliations:** ^1^ Department of Biomedical Physiology and Kinesiology, Simon Fraser University, Burnaby, BC, Canada; ^2^ Stanford Cardiovascular Institute, Stanford University, Stanford, CA, United States; ^3^ Department of Medicine, Stanford University, Stanford, CA, United States

**Keywords:** ARumenamide, loss-of-function, Brugada syndrome, Nav1.5, fenestrations, aromaticity, aliphaticity

## Abstract

**Background:** Most therapeutics targeting cardiac voltage-gated sodium channels (Nav1.5) attenuate the sodium current (I_Na_) conducted through the pore of the protein. Whereas these drugs may be beneficial for disease states associated with gain-of-function (GoF) in Nav1.5, few attempts have been made to therapeutically treat loss-of-function (LoF) conditions. The primary impediment to designing efficacious therapies for LoF is a tendency for drugs to occlude the Nav1.5 central pore. We hypothesized that molecular candidates with a high affinity for the fenestrations would potentially reduce pore block.

**Methods and Results:** Virtual docking was performed on 21 compounds, selected based on their affinity for the fenestrations in Nav1.5, which included a class of sulfonamides and carboxamides we identify as *ARumenamide (AR)*. Six *ARs*, AR-051, AR-189, AR-674, AR-802, AR-807 and AR-811, were further docked against Nav1.5 built on NavAb and rNav1.5. Based on the virtual docking results, these particular *AR*s have a high affinity for Domain III-IV and Domain VI-I fenestrations. Upon functional characterization, a trend was observed in the effects of the six *ARs* on I_Na_. An inverse correlation was established between the aromaticity of the *AR*’s functional moieties and compound block. Due to its aromaticity, AR-811 blocked I_Na_ the least compared with other aromatic *ARs*, which also decelerated fast inactivation onset. AR-674, with its aliphatic functional group, significantly suppresses I_Na_ and enhances use-dependence in Nav1.5. AR-802 and AR-811, in particular, decelerated fast inactivation kinetics in the most common Brugada Syndrome Type 1 and Long-QT Syndrome Type 3 mutant, E1784K, without affecting peak or persistent I_Na_.

**Conclusion:** Our hypothesis that LoF in Nav1.5 may be therapeutically treated was supported by the discovery of *ARs*, which appear to preferentially block the fenestrations. *ARs* with aromatic functional groups as opposed to aliphatic groups efficaciously maintained Nav1.5 availability. We predict that these bulkier side groups may have a higher affinity for the hydrophobic milieu of the fenestrations, remaining there rather than in the central pore of the channel. Future refinements of *AR* compound structures and additional validation by molecular dynamic simulations and screening against more Brugada variants will further support their potential benefits in treating certain LoF cardiac arrhythmias.

## Introduction

The cardiac voltage-gated sodium channel (Nav1.5) is responsible for electrical depolarization in cardiac tissue through the conduction of sodium current (I_Na_). Mutations in the *SCN5a* gene, encoding Nav1.5, result in loss-of-function (LoF) or gain-of-function (GoF) abnormalities in the protein, leading to an array of cardiac disorders (Dumaine et al., 1996; Antzelevitch et al., 2007; Makita et al., 2008; Gui et al., 2010; Biswas et al., 2019). The common clinical manifestation of LoF mutations is Brugada Syndrome Type 1, whereas Long-QT syndrome Type 3 is caused by GoF (Antzelevitch et al., 2005; Beaufort-Krol et al., 2005; Bankston et al., 2007). Both these arrhythmogenic syndromes increase the likelihood of sudden cardiac arrest in patients (Kaufman, 2009; Skinner et al., 2019). The most common Brugada Syndrome Type 1 and Long-QT Syndrome Type 3 mutant is E1784K (Makita et al., 2008; Peters et al., 2020).

Non-genetic factors common in disease states can also trigger LoF and GoF in Nav1.5 through the activation of multiple cellular signaling cascades that enhance intracellular kinases and cytosolic calcium (Song et al., 2001; Baartscheer et al., 2003; Wagner et al., 2006). Increased phosphorylation in Nav1.5 augments late or persistent I_Na_ and enhances entry into fast and slow inactivation (Wagner et al., 2006, 2011; Herren et al., 2013). An elevated sympathetic drive increases the heart rate and promotes rapid onset into fast inactivation in Nav1.5, prematurely terminating peak I_Na_. With time, channels also accumulate into slow inactivation, which lowers the available pool of Nav1.5 required to sustain normal heart rhythmicity (Ashpole et al., 2012; Herren et al., 2013).

To date, the major pharmaceuticals targeting Nav1.5 treat GoF. These agents, like ranolazine, phenytoin, and lidocaine, to name a few, generally block the central pore of Nav1.5 by interacting with residues in the inner vestibule (Balser et al., 1996a, 1996b; An et al., 1996; Antzelevitch, 2004; Sokolov et al., 2013; Abdelsayed et al., 2018). Very few attempts, however, have been made to target LoF. Toxins, such as batrachotoxin, were regarded as potential hits for treating LoF since they bind to Na_V_ and stabilize the channel in the open state (Du et al., 2011; Wang and Wang, 2014; Finol-Urdaneta et al., 2019; MacKenzie et al., 2022). Open-state stabilization, however, may produce other adverse side effects that further exacerbate the pathophysiology (MacKenzie et al., 2022). Inadequate targeting of LoF leaves behind a plethora of untreated Na_V_-related diseases like Brugada Syndrome Type 1.

Targeting other sites besides the pore regions in Nav1.5 is the most prudent approach in treating LoF to avoid blocking peak I_Na_. Rearrangement in the four fenestrations of Na_V_ has been implicated as an important pathway for lipophilic drug entry into the channel pore (Payandeh et al., 2011, 2012; Kaczmarski and Corry, 2014; Jiang et al., 2020). In 1977, Hille proposed that, unlike charged compounds, lipophilic compounds would enter the channel through these fenestrations (Hille, 1977). His prediction was correct, and structural and electrophysiological studies proved that bulky drugs, especially ones like flecainide elicit their state-dependent effects on Nav via the fenestrations (Kaczmarski and Corry, 2014; Gamal El-Din et al., 2018; Montini et al., 2018).

Comparison between the structural models of the closed and open Nav from *Arcobacter butzleri* (NavAb) and *Magnetococcus marinus* (NavMs) confirmed that at rest, drugs, especially lipophilic ones, have a high access into the channel through the dilated fenestrations and await the dilation of the central pore to elicit their pharmacological action (Payandeh et al., 2011; Martin and Corry, 2014; Montini et al., 2018). However, other drug-binding modes, such as during open-state or the inactivated-state, are not explained by this mechanism of action, although they may involve the fenestrations (Martin and Corry, 2014). Open-state drug binding is likely to occur via the open activation gate through the intracellular pathway (Lenaeus et al., 2017). Classic antiarrhythmics, such as benzocaine and lidocaine, have low thermodynamic stability in the fenestrations; thus, they rapidly move to the inner vestibule and bind to their receptor sites (Kaczmarski and Corry, 2014). Benzocaine’s binding site in NavAb is F203 that is a surrogate for a conserved phenylalanine found in Domain IV S6 of eukaryotic Nav (Boiteux et al., 2014).

The recently solved cryo-EM structure of rat Nav1.5 (rNav1.5) at 3.2–3.5 Å resolution was captured in a state in which all four voltage sensors were partially activated; hence, the channel was also partially inactivated. The four fenestrations were identified: Domain II-III fenestration was the largest compared to the others (Jiang et al., 2020). Flecainide associates with residues in the central cavity via Domain II-III fenestration. Other studies suggest that Domain III-IV fenestration, can also provide access for the drug (Nguyen et al., 2019); however, this fenestration was too narrow in this model.

A major caveat of the cyro-EM structure of rNav1.5 is that it does not fully depict fenestration size at rest, nor in activated or inactivated states. Crystal structures obtained of NavAb inactivated states are compatible with slow inactivation in eukaryotic Nav channels, since NavAb lacks the fast inactivation particle (Payandeh et al., 2011, 2012). Upon inactivation, the wild-type NavAb undergoes reshaping of the fenestrations where two opposing fenestrations dilate and the other two constrict (Payandeh et al., 2012).

The mammalian skeletal muscle voltage-gated sodium channel, Nav1.4, homology model built on NavAb, also confirmed the previous finding in that two fenestrations, Domains II-III and IV-I, are narrower than the adjacent two (Domain I-II and III-IV) and their radial size is determined mainly by isoleucine and phenylalanine residues in S5s (Kaczmarski and Corry, 2014). The cryo-EM structure solved for the American cockroach voltage-gated sodium channel (NavPas) contains only one small fenestration formed by the pore-forming segments in Domains III-IV (Shen et al., 2017). Using MD simulations, Tao and Corry (2022) have characterized the fenestrations in the Nav subtypes based on the recent published structures (Tao and Corry, 2022). In contrast to the results reported by Jiang et al. (2020) in Na_V_1.5, DI-II and DIII-IV fenestrations are dilated, and DII-III and DI-IV fenestrations are constricted (Tao and Corry, 2022).

Our hypothesis is based on the premise that compounds dock in the dilated fenestrations during activation, and the fenestrations are prevented from constricting during inactivation, thereby maintaining a dilated and unblocked central pore, thus maintaining I_Na_ conduction. In this study, we used virtual docking and whole-cell patch clamp to assess the efficacy of a new class of compounds, *ARumenamide (AR)*, that dock within the fenestrations, in an effort to target LoF. We screen high profile *ARumenamides* against E1784K to validate their effect in attenuating LoF.

## Materials and methods

### Homology modeling

Homology modeling was performed using the Swiss-Model server (swissmodel.expasy.org) (Bordoli et al., 2008). We generated various homology models of Nav1.5 using several templates of Na_V_ crystallized or solved via cryo-EM. Not all were ideal for docking *ARumenamide* compounds since many of their fenestrations were too constricted or obscure from targeting.

The pre-activated bacterial crystal structure of NavAb (2.7 Å resolution) was used as a template against the Nav1.5 sequence. Modeling was done according to the protocol established by Bordoli et al. (2008). PyMOL-pdb viewer was used for optimization and structure visualization. The cryo-EM structure of rNav1.5 at 3.2–3.5 Å resolution was also used as a template against Nav1.5. Domain II-III fenestration was the largest compared to other fenestrations (Jiang et al., 2020).

### Docking

Virtual docking was preferentially performed against the Nav1.5-NavAb model, which has two accessible fenestrations (Payandeh et al., 2012). The Nav1.5 homology model built on NavAb (Nav1.5-NavAb) was docked against the ZINC free database using DOCK Blaster server (blaster.docking.org) (Morris et al., 2009). The highest 20 hits (Table 1), selected based on their binding affinity (kcal/mol) to Nav1.5-NavAb, were then virtually screened against Nav1.5-NavAb using AutoDock4 (Morris et al., 2009). PyMOL-pdb viewer was used for optimization and visualization of the auto-docking results.

**TABLE 1:** Chemical name and formula

Name	Formula
AR-787	N-(1H-benzimidazol-2-yl)-4-methyl-2-pyrrol-1-yl-thiazole-5-carboxamide
AR-138	N-(4-ethylphenyl)-2-oxo-benzimidazole-5-sulfonamide
AR-051	N-(2-furylmethyl)-2,3-dioxo-quinoxaline-6-carboxamide
AR-946	N-(3-imidazol-1-ylpropyl)-2,3-dioxo-quinoxaline-6-carboxamide
AR-634	N-(2-cyclohexen-1-ylethyl)-2,3-dioxo-quinoxaline-6-carboxamide
AR-058	6-(4-methylpiperazin-1-yl)sulfonylquinoxaline-2,3-dione
AR-674	N-allyl-2-oxo-benzimidazole-5-sulfonamide
AR-591	N-(m-tolyl)-2,3-dioxo-quinoxaline-6-sulfonamide
AR-133	N-(3-ethylphenyl)-2-oxo-benzimidazole-5-sulfonamide
AR-538	N-ethyl-N-(4-fluorophenyl)-2-oxo-benzimidazole-5-sulfonamide
AR-189	N-[(2S)-2-(dimethylamino)-2-(2-furyl)ethyl]-2,3-dioxo-quinoxaline-6-carboxamide
AR-769	N-[2-(4-methoxyphenyl)ethyl]-2-oxo-benzimidazole-5-sulfonamide
AR-949	N-methyl-N-[(2-methyl-3-furyl)methyl]-2-oxo-benzimidazole-5-sulfonamide
AR-310	5-[2-[2-furylmethyl (methyl)amino]acetyl]benzimidazol-2-one
AR-847	N-(1-methylpyrazol-3-yl)-2,3-dioxo-quinoxaline-6-sulfonamide
AR-792	N-(2-chloro-4-methyl-phenyl)-2-oxo-benzimidazole-5-sulfonamide
AR-802	N-(3-methoxyphenyl)-2-oxo-benzimidazole-5-sulfonamide
AR-807	N-(2-ethylphenyl)-2-oxo-benzimidazole-5-sulfonamide
AR-811	N-(4-fluoro-2-methyl-phenyl)-2-oxo-benzimidazole-5-sulfonamide
AR-812	N-[(2-methoxyphenyl)methyl]-2-oxo-benzimidazole-5-sulfonamide

**FIGURE 1:**
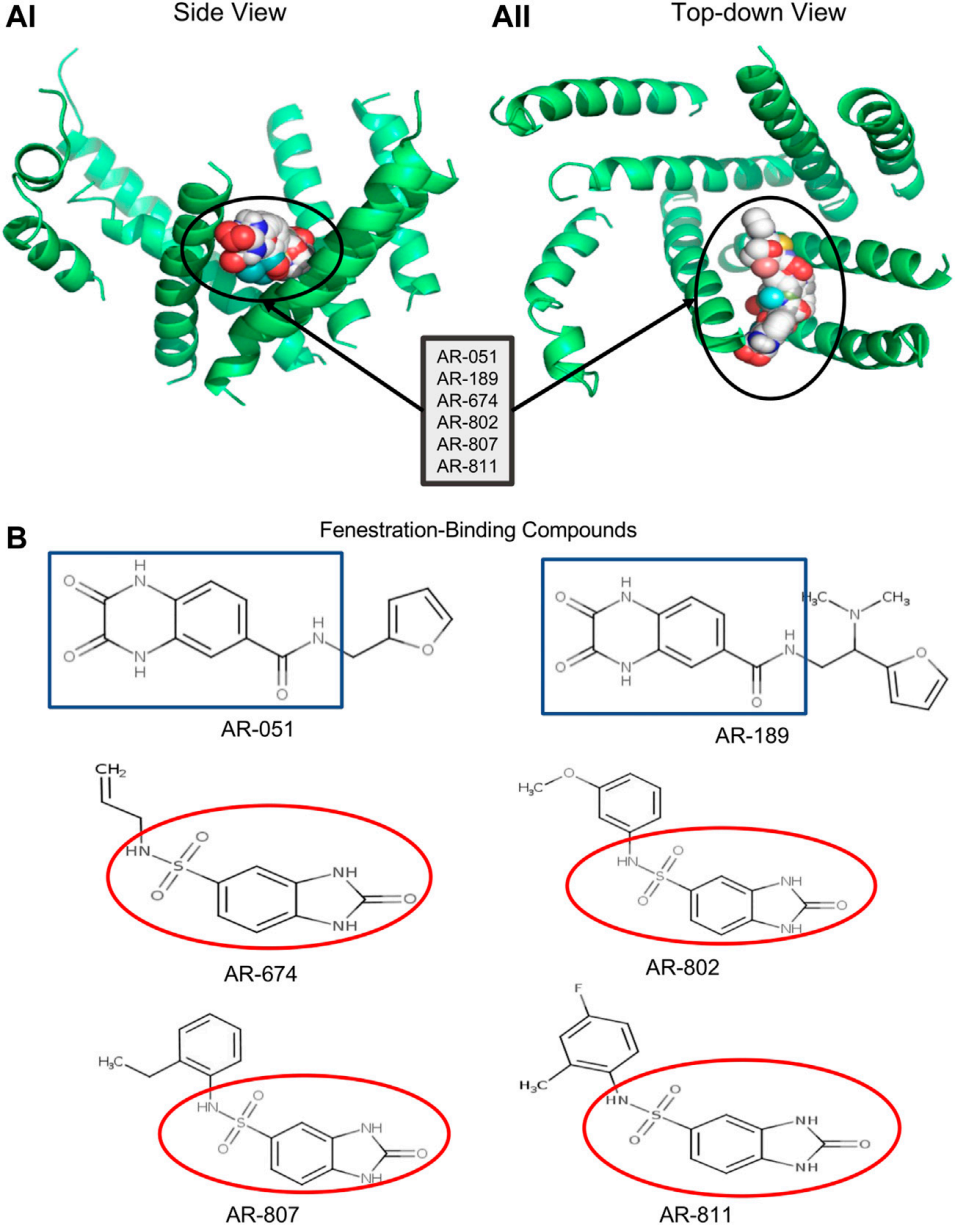
Docking ARumenamide. **(AI, AII)** display the virtual docking results for *ARumenamide* compounds screened against Nav1.5 -NavAb from the side and top-down views, respectively. The highest binding affinity mode is shown for the six *ARumenamide* compounds: AR-051; AR-189; AR-674; AR-802; AR-807; and AR-811. All these compounds have a favorable affinity to Domain III-IV fenestration (circled). The compounds are shown as spherical representations. **(B)** shows the chemical structure of the docked *ARumenamide* compounds. The common backbone is either squared (blue) if it contains a carboxamide or circled if it contains sulfonamide (red). The functional groups (non-squared and non-circled) are the key moieties and determinants of the *ARumenamide’s* efficacy.

Given the unique features of the Nav1.5-NavAb compared to other homology models, we selected six *ARs* for functional characterization using patch clamp electrophysiology. We based this decision on each *AR* compound’s affinity and probability of adhering to the fenestrations (Figure 1). We subsequently docked the same compounds against the rNav1.5 channel (Jiang et al., 2020) as a means of determining the potential binding state of the compounds during fast inactivation. In either channels (Nav1.5-NavAb or rNav1.5), compounds were screened against individual fenestrations: Domain I-II, Domain II-III, Domain III-IV, and Domain IV-I. Finally, the compounds were screened against the complete channel, with the four domains intact.

### Compounds

The six chosen *ARumenamide* compounds (Figure 1) can be generally categorized into carboxamides and sulfonamides: AR-051, AR-189, AR-674, AR-802, AR-807 and AR-811. *ARumenamide* compounds were purchased from MolPort SIA (Riga, Latvia) in powder form. They were dissolved in 100% DMSO to create a stock solution of 50 mM and diluted with external solution to the desired final concentration.

AR-051 (MolPort-002-662-051/ZINC38767171/STK615542)

AR-189 (MolPort-004-279-189/ZINC40014265/Z26997243)

AR-674 (MolPort-001-911-674/ZINC39699427/STK742976)

AR-802 (MolPort-010-669-802/ZINC64470729/G801-0062)

AR-807 (MolPort-010-669-807/ZINC64470737/G801-0091)

AR-811 (MolPort-010-669-811/ZINC64470745/G801-0104)

Vehicle (0.05% DMSO) controls were often performed to enable correction for any compound-independent decrease of currents. Since Na_V_1.5 is TTX-resistant, we used lidocaine to confirm the *ARumenamide* effects observed in E1784K.

### Cell culture

HEK293 cells were grown at pH 7.4 in a DMEM (1x) nutrient medium (Life Technologies, NY, United States), supplemented with 10% FBS and maintained in a humidified environment at 37°C with 5% CO_2_. The α-subunits (WT or E1784K) were transiently co-transfected with the β1 subunit and green fluorescent protein, eGFP (1.50 μg: 0.75 μg: 1.50 μg, respectively). The cDNA mixture was then allowed to incubate with the HEK293 cells before plating on coverslips.

### Electrophysiology

Whole-cell patch clamp recordings were performed in extracellular solution containing (mM): 140 NaCl, 4 KCl, 2 CaCl_2_, 1 MgCl_2_, and 10 HEPES (pH 7.4 adjusted with CsOH). Solutions were titrated with CsOH to pH 7.4. Pipettes were fabricated with a P-1000 puller using borosilicate glass (Sutter Instruments, CA, United States), dipped in dental wax to reduce capacitance, then thermally polished to a resistance of 1.0–1.5 MΩ. Low resistance electrodes were used to minimize series resistance between pipette and intracellular solution resulting in typical access resistances of 3.5 MΩ or less, thereby minimizing voltage measurement error. Pipettes were filled with intracellular solution (in mM): 130 CsF, 10 NaCl, 10 HEPES, and 10 EGTA titrated to pH 7.4.

All recordings were made using an EPC-9 patch-clamp amplifier (HEKA Elektronik, Lambrecht, Germany) digitized at 20 kHz using an ITC-16 interface (HEKA Elektronik, Lambrecht, Germany). Data were acquired and low-pass-filtered (5 kHz) using PatchMaster/FitMaster software (HEKA Elektronik, Lambrecht, Germany) running on an Apple iMac (Apple Computer, Cupertino, CA). Leak subtraction was performed online using a P/4 procedure. Bath solution temperature was controlled using a Peltier device driven by a TC-10 Temperature Controller (Dagan, Minneapolis, MN), and maintained at 37°C. When screening the effects of AR-802 and AR-811 on E1784K, bath solution temperature was maintained at 22°C since it was difficult to record at higher temperatures due to the inherent instability of cells.

The six *ARumenamide* compounds chosen for biophysical characterization were screened against wild type (WT) and E1784K Nav1.5 by bath perfusion following an initial series of voltage clamp protocols (below).

### Voltage clamp protocols

*Conductance* was measured by a protocol that depolarized the membrane from -100 mV to +80 mV in increments of +5 mV for 19 ms. Prior to the test pulse, channels were allowed to recover from fast inactivation at −130 mV for 197 ms. Channel conductance was calculated from peak I_Na_

$$G_{Na}=I_{Na}/V-E_{Rev}$$ (1)

where G_Na_ is sodium channel conductance, I_Na_ is peak sodium current in response to the command potential V, and E_Rev_ is the reversal potential. Calculated values for conductance were fitted with the Boltzmann function:

$$G/G_{\max}=1/\left(1+\exp\left[-{ze}_{0}\left[V_{M}-V_{1/2}\right]/kT\right]\right)$$ (2)

where G/G_max_ is the normalized conductance amplitude, V_M_ is the command potential, z is the apparent valency, e_0_ is the elementary charge, V_1/2_ is the midpoint voltage, k is the Boltzmann constant, and T is temperature in °K.

*Fast Inactivation Onset:* At voltages greater than −50 mV, the fast inactivation τ values were calculated from the mono-exponential fit to the decay of sodium current.

$${I=I}_{ss}+\alpha \exp\left(-\left(t-t_{0}\right)/\tau \right)$$ (3)

where I is current amplitude, I_ss_ is the plateau amplitude, α is the amplitude at time 0 for time constant τ, and t is time.

*Persistent I*_*Na*_ *current* was measured by holding the potential at -110 mV, −90 mV, or −70 mV for 1 s followed by a depolarization to 0 mV for 200 ms and repolarization to −110 mV for 50 ms. The fast inactivation τ values were also calculated from the mono-exponential fit to the decay of sodium current measured in this protocol.

*Use-Dependence* was used to indirectly measure slow inactivation (SI), which is a physiologically relevant protocol. The protocol includes a series of 110 ms depolarizing pulses to 0 mV followed by a –90 mV recovery pulse for 55 ms at a frequency 6 Hz. With repetitive depolarizations, Nav1.5 channels accumulate into slow inactivation. Normalized current amplitude as a function of time was fit with a double exponential.

$${I=I}_{ss}+\alpha_{1}\exp\left(-t/\tau_{1}\right)+\alpha_{2}\exp\left(-t/\tau_{2}\right)$$ (4)

where I is current amplitude, I_ss_ is the plateau amplitude, α_1_ and α_2_ are the amplitudes at time 0 for time constants τ_1_ and τ_2_, and t is time.

### Statistical analysis

Statistical analysis was performed using a one-factor completely randomized design ANOVA followed by a post hoc Tukey test. Our statistical model was a full factorial in which all the factors were allowed to interact together. Data are shown as mean ± SEM. Statistical significance was considered at *p* < 0.05.

### Intellectual property

*ARumenamides* described here are under IP protection (Publication Number WO/2020/161606; International Application No. PCT/IB 2020/050853).

## Results

### Docking in the fenestrations

Six *ARumenamide* hits, AR-051, AR-189, AR-674, AR-802, AR-807, and AR-811, are shown in Figure 1 docked, in their highest affinity, to the Domain III-IV fenestration in Nav1.5-NavAb. In addition, there were states in which the compounds interacted with lower affinities with other channel regions, including other fenestrations, the central pore, the pore-forming and voltage-sensing domains. The existence of additional interaction sites is important when interpreting our electrophysiological findings. The *ARumenamides* displayed the same binding affinity (in kcal/mol) whether they were docked against the individual fenestrations or the entire channel.

None of the six *ARumenamide* hits screened against rNav1.5 interacted with Domain I–II and Domain II–III fenestrations (Table 2). The compounds, however, had a high affinity for Domain III–IV and Domain IV-I fenestrations. When docked only against Domain III–IV fenestration, AR-674, AR-807, and AR-811 displayed the highest binding affinities compared to the other compounds. All compounds displayed high affinity to Domain IV-I fenestration in rNav1.5 (Table 2). When all four domains were reannealed together to form an intact channel, virtual docking confirmed that most compounds preferentially interacted with Domain IV–I fenestration compared to Domain III–IV fenestration (Table 2).

**TABLE 2:** Binding sites and affinities for compounds docked against rNav1.5

	Highest affinity binding mode (kcal/mol)									
	DI-DII		DII-DIII		DIII-DIV		DIV-DI		Whole channel	
**AR-051**	−7.0	VSD	−7.5	VSD	−6.9	OUT VES	−7.7	FEN	−8.0	DIV-DI FEN
**AR-189**	−6.6	VSD	−6.4	VSD	−6.9	VSD	−6.8	FEN	−7.0	DIV-DI FEN
**AR-674**	−8.3	VSD	−7.4	OUT VES	−7.7	FEN	−8.0	FEN	−8.6	DIV-DI FEN
**AR-802**	−7.3	VSD	−7.3	VSD	−7.9	VSD	−8.6	FEN	−9.0	DIV-DI FEN
**AR-807**	−7.6	VSD	−7.5	VSD	−8.7	FEN	−8.1	FEN	−8.8	DIV-DI FEN
**AR-811**	−8.1	VSD	−7.9	VSD	−8.7	FEN	−8.8	FEN	−9.0	DIV-DI FEN

*VSD, Voltage-Sensing Domain.

*OUT VES, outer vestibule.

*INN VES, inner vestibule.

*FEN, fenestration.

### Screening the six ARumenamide compounds on wild-type Na_V_1.5

**FIGURE 2:**
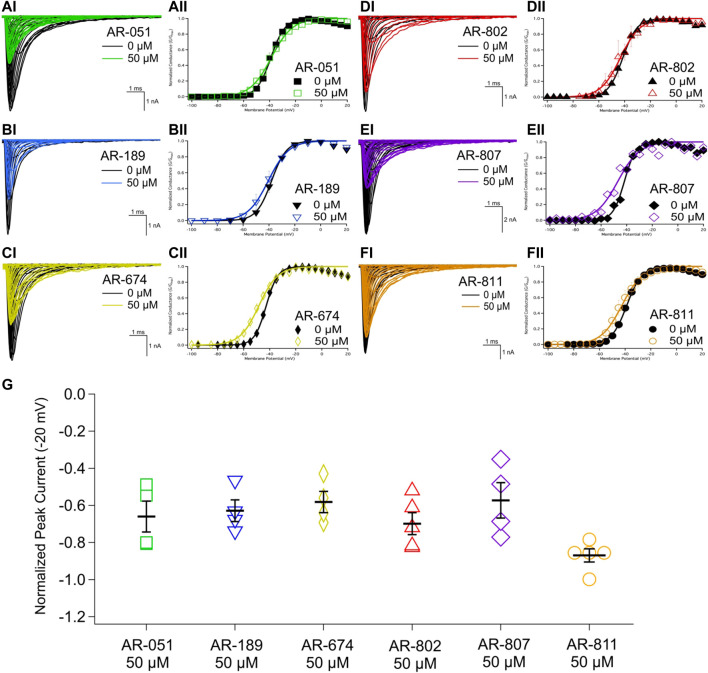
Peak I_NA_ and conductance. **(AI-FI)** show representative traces of peak I_Na_ in vehicle (black) and compound treatment at 50 µM (varied colors). **(AII-FII)** shows the voltage-dependence of conductance fit with a Boltzmann curve for vehicle (black) and compound treatment at 50 µM (varied colors). **(G)** shows a box-plot for compound-induced shifts in normalized peak I_Na_ (normalized to control) at −20 mV. Statistical significance is reported in Table 3.

**FIGURE 3:**
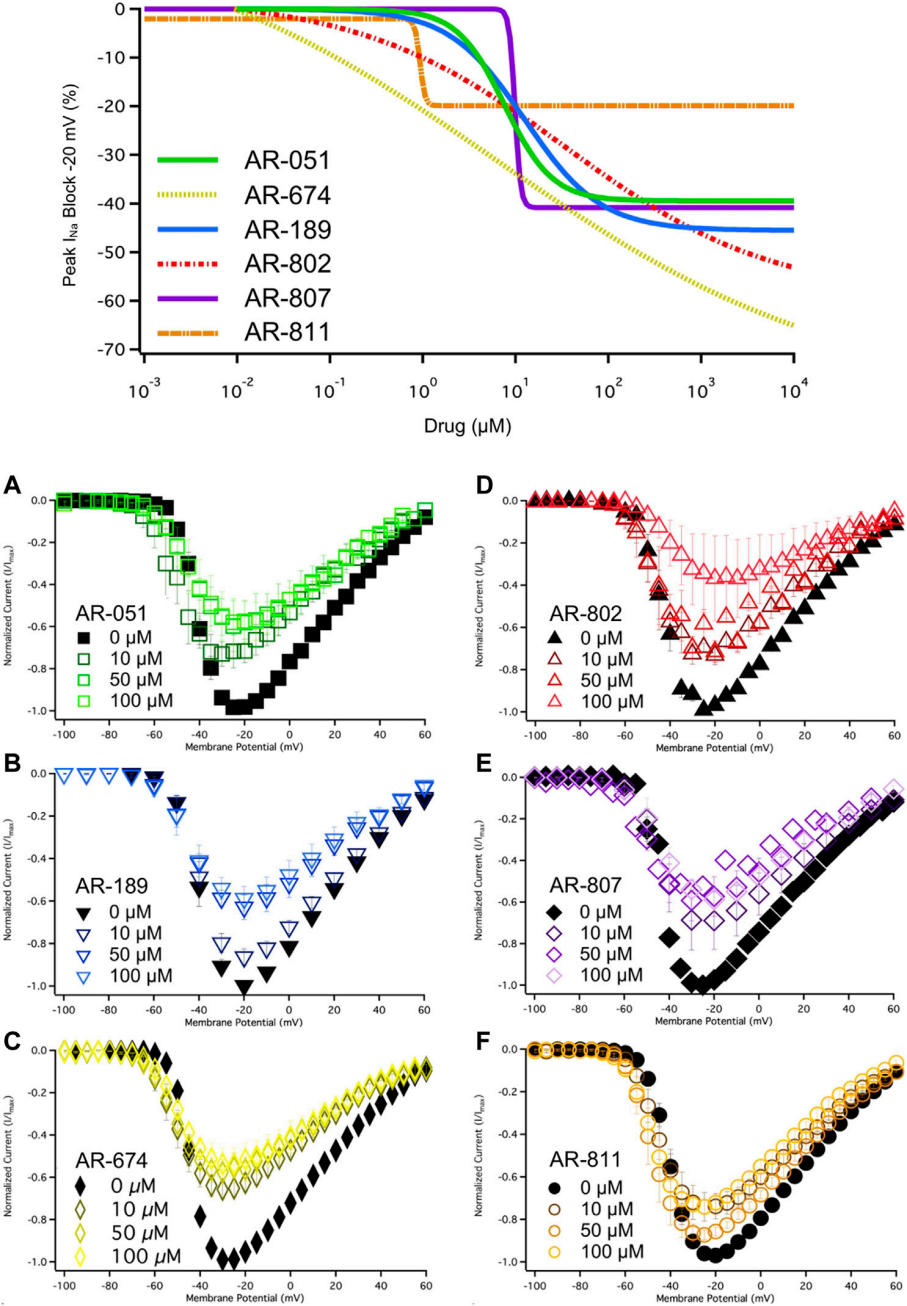
Dose-Response and current-voltage relationships. A dose-response curve was fitted to the compound concentrations for peak INa block at –20 mV, generating hill curves shown at the top of the figure. Parameters from the Hill function curve are reported in Table 4. **(A-F)** show the current-voltage relationship for vehicle (black) and compound treatment between 0–100 μMs (varied colors). Statistical significance is reported in Table 3.

All six *ARumenamide* compounds assessed virtually were functionally screened against wild type Nav1.5 using whole-cell patch clamp. The compounds displayed different affinities to block peak I_Na_ at −20 mV (Figure 2AI-FI and Table 3). AR-674 blocked peak I_Na_ at −20 mV by 34% (*p* < 0.05) at 10 µM and progressively more with higher compound concentrations. AR-051, AR-189, AR-802, and AR-807 all significantly blocked peak I_Na_ at −20 mV at concentrations of 50 µM and higher. AR-811 was the only compound that did not block peak I_Na_ within the concentrations tested (1–100 µM) (Figure 3 and Table 3). A dose-response curve is shown for peak I_Na_ block at −20 mV in Figure 3 along with the Hill function parameters in Table 4. The trends observed in these parameters confirm that AR-674 is the most potent in blocking peak I_Na_ compared to the rest of the compounds.

**TABLE 3:** Peak I_NA_ and Conductance.

Treatment	Peak I_Na_ (−20 mV)	% I_Na_ block (−20 mV)	Conductance midpoint GV-V_1/2_	Conductance slope GV-z	N
**AR-051 (0 µM)**	−0.95 ± 0.04	0.00	−42.12 ± 3.00	5.47 ± 0.28	7
**AR-051 (10 µM)**	−0.72 ± 0.06	−24.21	−45.58 ± 4.20	4.19 ± 0.42	4
**AR-051 (50 µM)**	−0.59 ± 0.11*	−37.89*	−39.12 ± 1.76	3.40 ± 0.21*	4
**AR-051 (100 µM)**	−0.58 ± 0.10*	−38.95*	−40.47 ± 5.25	3.33 ± 0.32*	3
**AR-189 (0 µM)**	−1.00 ± 0.01	0.00	−38.04 ± 1.57	5.13 ± 0.39	6
**AR-189 (10 µM)**	−0.80 ± 0.06	−20.00	−38.44 ± 1.60	4.47 ± 0.39	6
**AR-189 (50 µM)**	−0.63 ± 0.06*	−37.00*	−40.77 ± 3.17	3.79 ± 0.07	4
**AR-189 (100 µM)**	−0.59 ± 0.06*	−41.00*	−40.62 ± 4.48	3.67 ± 0.15	3
**AR-674 (0 µM)**	−1.00 ± 0.00	0.00	−42.74 ± 0.92	6.61 ± 0.52	7
**AR-674 (10 µM)**	-0.66 ± 0.04*	−34.00*	−47.63 ± 2.20	4.46 ± 0.23*	6
**AR-674 (50 µM)**	−0.58 ± 0.06*	−42.00*	−48.47 ± 1.63	3.87 ± 0.32*	4
**AR-674 (100 µM)**	−0.53 ± 0.07*	−47.00*	−46.11 ± 0.48	3.72 ± 0.15*	3
**AR-802 (0 µM)**	−0.97 ± 0.02	0.00	−39.21 ± 1.63	5.21 ± 0.24	11
**AR-802 (10 µM)**	−0.75 ± 0.05	−22.68	−41.23 ± 1.87	4.51 ± 0.19	8
**AR-802 (50 µM)**	−0.72 ± 0.06*	−25.77*	−42.19 ± 3.27	4.15 ± 0.27	6
**AR-802 (100 µM)**	−0.60 ± 0.26*	−38.14*	−36.61 ± 3.15	3.66 ± 0.79*	3
**AR-807 (0 µM)**	−0.98 ± 0.01	0.00	−42.14 ± 0.81	5.64 ± 0.48	6
**AR-807 (10 µM)**	−0.76 ± 0.13	−22.45	−43.56 ± 2.51	4.54 ± 0.47	5
**AR-807 (50 µM)**	−0.58 ± 0.10*	−40.82*	−44.22 ± 2.13	3.38 ± 0.32	4
**AR-807 (100 µM)**	−0.25 ± 0.01*	−74.49*	−40.48 ± 0.01	2.60 ± 0.01*	3
**AR-811 (0 µM)**	−0.97 ± 0.01	0.00	−38.70 ± 1.37	5.52 ± 0.28	17
**AR-811 (10 µM)**	−0.74 ± 0.06	−23.71	−42.72 ± 2.57	4.56 ± 0.45	7
**AR-811 (50 µM)**	−0.85 ± 0.08	−12.37	−42.15 ± 2.97	4.57 ± 0.54	6
**AR-811 (100 µM)**	−0.73 ± 0.04	−24.74	−43.95 ± 1.89	4.20 ± 0.42	6

*p < 0.05 vs. 0** **µM (AR, compound).

**TABLE 4:** Hill function parameters for peak I_NA_ and use-dependence block.

	Peak I_Na_ block (−20 mV)		Use-dependence Block (%)	
	Rate	IC_50_ (µM)	Rate	IC_50_ (µM)
**AR-051**	1.70	7.61	0.15	0.00149
**AR-189**	1.06	12.56	0.09	0.00001
**AR-674**	0.24	6.45	0.53	245.78
**AR-802**	0.39	34.63	4.16	7.61
**AR-807**	14.37	9.86	0.57	2199.30
**AR-811**	16.06	0.93	0.34	101.98

The voltage-dependence of conductance was assessed using a series of depolarizing test pulses. None of the *ARumenamide* compounds affected the midpoint potential value of conductance (Figure 2AII-FII and Table 3). AR-674 was highly potent (at 10 µM) in decreasing voltage-sensitivity of conductance as measured from the conductance slope (Table 3). Other compounds like AR-051 significantly decreased (*p* < 0.05) the conductance slope at 50 µM and higher. While AR-802 and AR-807 significantly decreased (*p* < 0.05) the conductance slope at 100 µM (Table 3).

**FIGURE 4:**
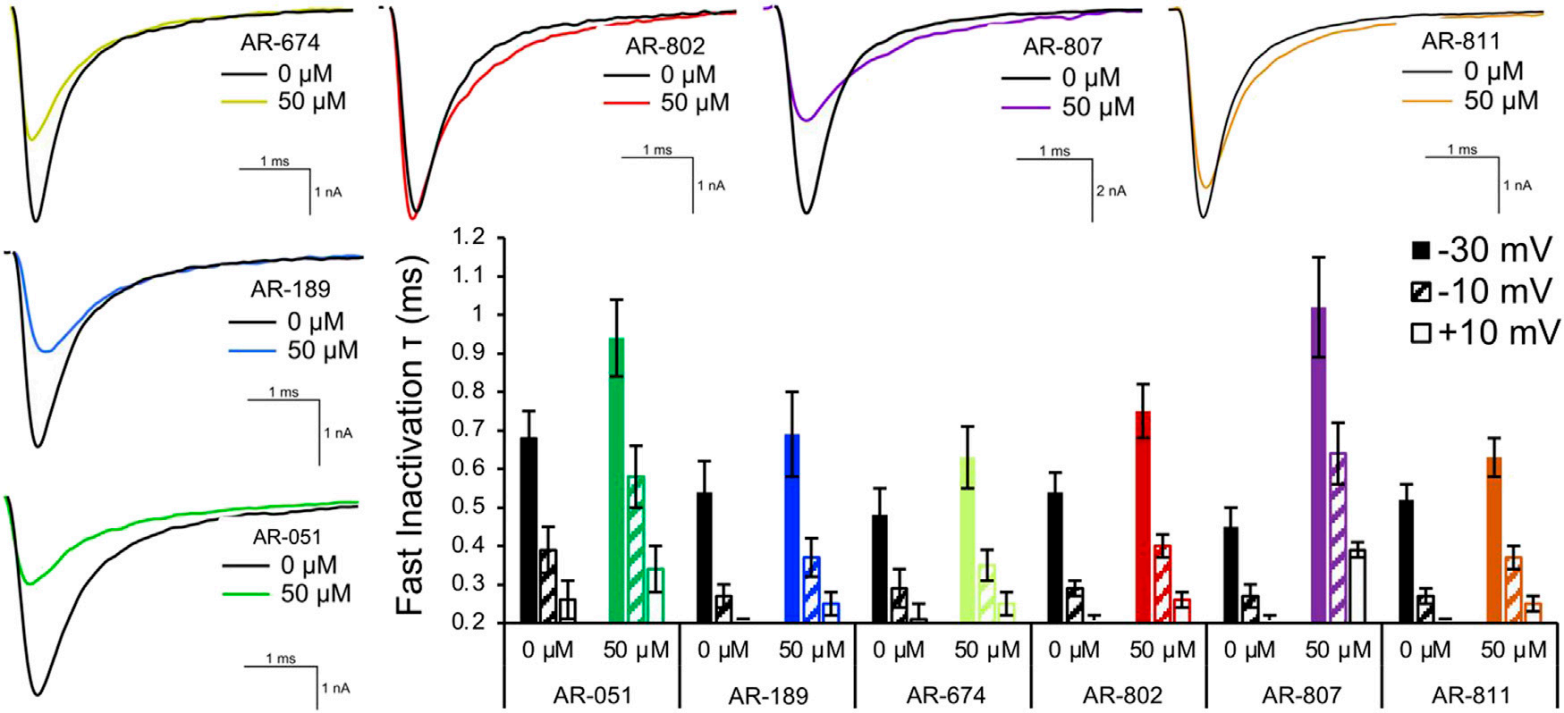
Fast inactivation onset. Representative traces are shown for vehicle (black) and compound treatment (varied colors) at –20 mV. The bar graph displays the effects of the compound (50 μM) on the fast inactivation time constant (τ in milliseconds) at –30 mV, –10 mV, and +10 mV. Statistical significance is reported in Table 5.

Open-state fast inactivation was measured at voltages greater than -50 mV (Figure 4). The compounds did not induce significant shifts in fast inactivation kinetics (*p* > 0.05) at −50 mV. However, AR-802 and AR-807 slowed the onset of fast inactivation (higher τ - Table 5) at 50 µM and higher. AR-807 was more potent than AR-802 in slowing fast inactivation kinetics at −30 mV, −10 mV, and +10 mV as low as 10 µM (Table 5). AR-051 was less effective in slowing fast inactivation kinetics and had only a subtle effect on τ at −30 mV at 100 µM. Similarly, AR-811 was only effective at 100 µM in decelerating fast inactivation at −30 mV, −10 mV, and +10 mV (Table 5).

**TABLE 5:** Fast inactivation onset.

Treatment	FI -50 mV (ms)	FI -30 mV (ms)	FI -10 mV (ms)	FI +10 mV (ms)	N
**AR-051 (0 µM)**	2.45 ± 0.30	0.68 ± 0.07	0.39 ± 0.06	0.26 ± 0.05	7
**AR-051 (10 µM)**	1.70 ± 0.33	0.71 ± 0.12	0.39 ± 0.06	0.25 ± 0.05	4
**AR-051 (50 µM)**	1.79 ± 0.15	0.94 ± 0.10	0.58 ± 0.08	0.34 ± 0.06	4
**AR-051 (100 µM)**	1.92 ± 0.38	1.05 ± 0.21*	0.60 ± 0.13	0.41 ± 0.11	3
**AR-189 (0 µM)**	3.26 ± 0.96	0.54 ± 0.08	0.27 ± 0.03	0.19 ± 0.02	6
**AR-189 (10 µM)**	2.98 ± 0.84	0.63 ± 0.07	0.33 ± 0.03	0.23 ± 0.02	6
**AR-189 (50 µM)**	2.18 ± 0.70	0.69 ± 0.11	0.37 ± 0.05	0.25 ± 0.03	4
**AR-189 (100 µM)**	2.17 ± 0.73	0.69 ± 0.17	0.37 ± 0.09	0.26 ± 0.07	3
**AR-674 (0 µM)**	1.82 ± 0.23	0.48 ± 0.07	0.29 ± 0.05	0.21 ± 0.04	7
**AR-674 (10 µM)**	1.32 ± 0.24	0.54 ± 0.09	0.31 ± 0.04	0.21 ± 0.03	6
**AR-674 (50 µM)**	1.46 ± 0.09	0.63 ± 0.08	0.35 ± 0.04	0.25 ± 0.03	4
**AR-674 (100 µM)**	1.61 ± 0.18	0.72 ± 0.04	0.40 ± 0.03	0.26 ± 0.02	3
**AR-802 (0 µM)**	2.24 ± 0.46	0.54 ± 0.05	0.29 ± 0.02	0.20 ± 0.02	12
**AR-802 (10 µM)**	1.67 ± 0.21	0.70 ± 0.14	0.40 ± 0.08	0.26 ± 0.04	8
**AR-802 (50 µM)**	1.97 ± 0.29	0.75 ± 0.07*	0.40 ± 0.03	0.26 ± 0.02	7
**AR-802 (100 µM)**	1.71 ± 0.22	0.76 ± 0.09*	0.39 ± 0.02	0.23 ± 0.03	4
**AR-807 (0 µM)**	1.74 ± 0.31	0.45 ± 0.05	0.27 ± 0.03	0.20 ± 0.02	6
**AR-807 (10 µM)**	1.37 ± 0.31	0.66 ± 0.09	0.41 ± 0.07	0.31 ± 0.06*	5
**AR-807 (50 µM)**	1.88 ± 0.27	1.02 ± 0.13*	0.64 ± 0.08*	0.39 ± 0.02*	4
**AR-807 (100 µM)**	1.82 ± 0.00	0.96 ± 0.01*	0.66 ± 0.01*	0.49 ± 0.01*	3
**AR-811 (0 µM)**	2.67 ± 0.38	0.52 ± 0.04	0.27 ± 0.02	0.19 ± 0.02	17
**AR-811 (10 µM)**	2.12 ± 0.34	0.66 ± 0.04	0.37 ± 0.03	0.27 ± 0.03	7
**AR-811 (50 µM)**	1.75 ± 0.34	0.63 ± 0.05	0.37 ± 0.03	0.25 ± 0.02	8
**AR-811 (100 µM)**	1.79 ± 0.20	0.81 ± 0.12*	0.45 ± 0.07*	0.28 ± 0.04*	6

*p < 0.05 vs. 0 µM (AR, compound).

**FIGURE 5:**
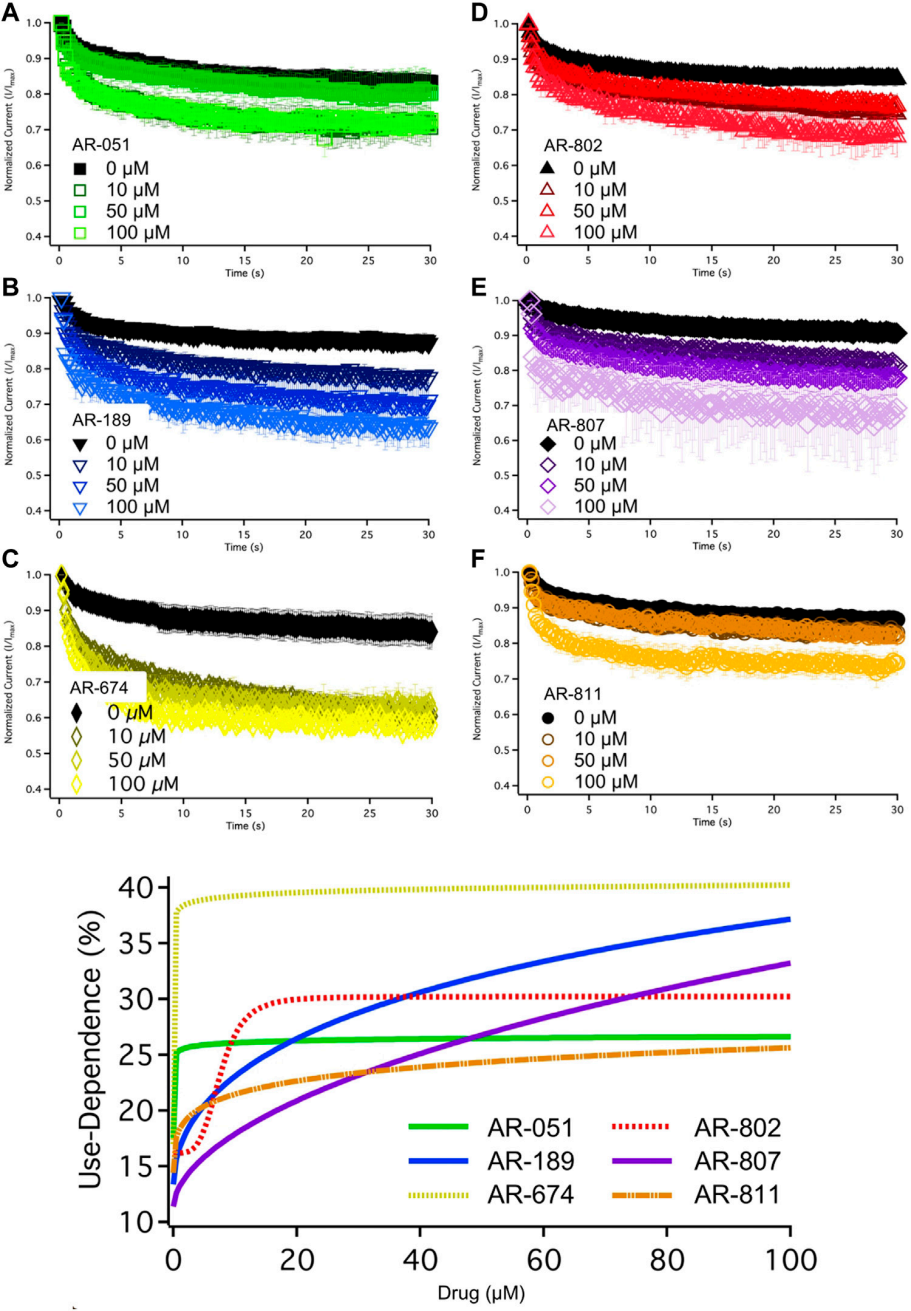
Use-Dependence. Nav1.5 use-dependence is shown for vehicle (black) and compound treatment (varied colors). A dose-response curve was fitted to the compound concentrations for use-dependence (%), generating hill curves shown at the bottom of the figure. Statistical significance is reported in Table 6.

The six *ARumenamide* compounds displayed similar trends in their effect on use-dependent decrease of current amplitude as they did with peak I_Na_ block (Figure 5). AR-674 significantly enhanced (*p* < 0.05) use-dependence in Nav1.5 at 10 µM and higher concentrations (y_0_ - Table 6). AR-189 also enhanced use-dependence but only at 50 µM and higher (Table 6). The rest of the compounds, namely AR-051, AR-802, AR-807, and AR-811 did not induce a significant on use-dependence. No compound-induced shifts were observed in t_1_ and t_2_, the parameters of the double-exponential fit to use-dependence (Table 6). The dose-response curve in Figure 5 shows the effect of AR-674 to profoundly increase Nav1.5 use-dependence (IC_50_ values and rates of Hill curves - Table 4).

**TABLE 6:** Use-dependence.

Treatment	y_0_	t_1_ (s)	t_2_ (s)	N
**AR-051 (0 µM)**	0.83 ± 0.04	2.25 ± 0.78	10.30 ± 1.01	12
**AR-051 (10 µM)**	0.72 ± 0.06	0.38 ± 0.09	7.21 ± 1.73	7
**AR-051 (50 µM)**	0.79 ± 0.04	0.86 ± 0.30	10.76 ± 3.05	5
**AR-051 (100 µM)**	0.72 ± 0.03	0.39 ± 0.09	9.40 ± 3.01	4
**AR-189 (0 µM)**	0.87 ± 0.02	0.84 ± 0.19	13.53 ± 4.20	7
**AR-189 (10 µM)**	0.77 ± 0.02	2.52 ± 2.01	9.45 ± 2.73	6
**AR-189 (50 µM)**	0.69 ± 0.06*	0.65 ± 0.13	12.48 ± 1.70	5
**AR-189 (100 µM)**	0.63 ± 0.06*	0.36 ± 0.02	11.66 ± 3.44	3
**AR-674 (0 µM)**	0.84 ± 0.05	1.65 ± 0.47	14.57 ± 3.96	8
**AR-674 (10 µM)**	0.52 ± 0.08*	5.27 ± 4.29	22.38 ± 3.84	7
**AR-674 (50 µM)**	0.63 ± 0.05*	0.52 ± 0.15	5.13 ± 0.52	2
**AR-674 (100 µM)**	0.68 ± 0.08*	1.20 ± 0.74	10.20 ± 6.21	3
**AR-802 (0 µM)**	0.84 ± 0.02	1.03 ± 0.27	8.01 ± 1.21	15
**AR-802 (10 µM)**	0.74 ± 0.05	1.74 ± 0.76	14.81 ± 3.14	10
**AR-802 (50 µM)**	0.69 ± 0.06	0.87 ± 0.25	18.67 ± 5.92	9
**AR-802 (100 µM)**	0.72 ± 0.08	0.44 ± 0.10	9.85 ± 5.43	3
**AR-807 (0 µM)**	0.90 ± 0.02	4.22 ± 1.42	22.28 ± 6.28	8
**AR-807 (10 µM)**	0.81 ± 0.03	2.25 ± 1.64	17.61 ± 5.07	6
**AR-807 (50 µM)**	0.76 ± 0.05	1.44 ± 0.92	18.07 ± 4.82	5
**AR-807 (100 µM)**	0.66 ± 0.11	0.39 ± 0.15	13.14 ± 1.02	2
**AR-811 (0 µM)**	0.86 ± 0.02	2.72 ± 1.02	18.17 ± 3.90	16
**AR-811 (10 µM)**	0.79 ± 0.05	1.48 ± 0.44	22.35 ± 8.39	7
**AR-811 (50 µM)**	0.78 ± 0.05	7.34 ± 6.79	21.85 ± 6.49	7
**AR-811 (100 µM)**	0.74 ± 0.03	0.57 ± 0.18	11.61 ± 5.38	5

*p < 0.05 vs. 0 µM (AR, compound).

### Screening AR-802 and AR-811 on E1784K

**FIGURE 6:**
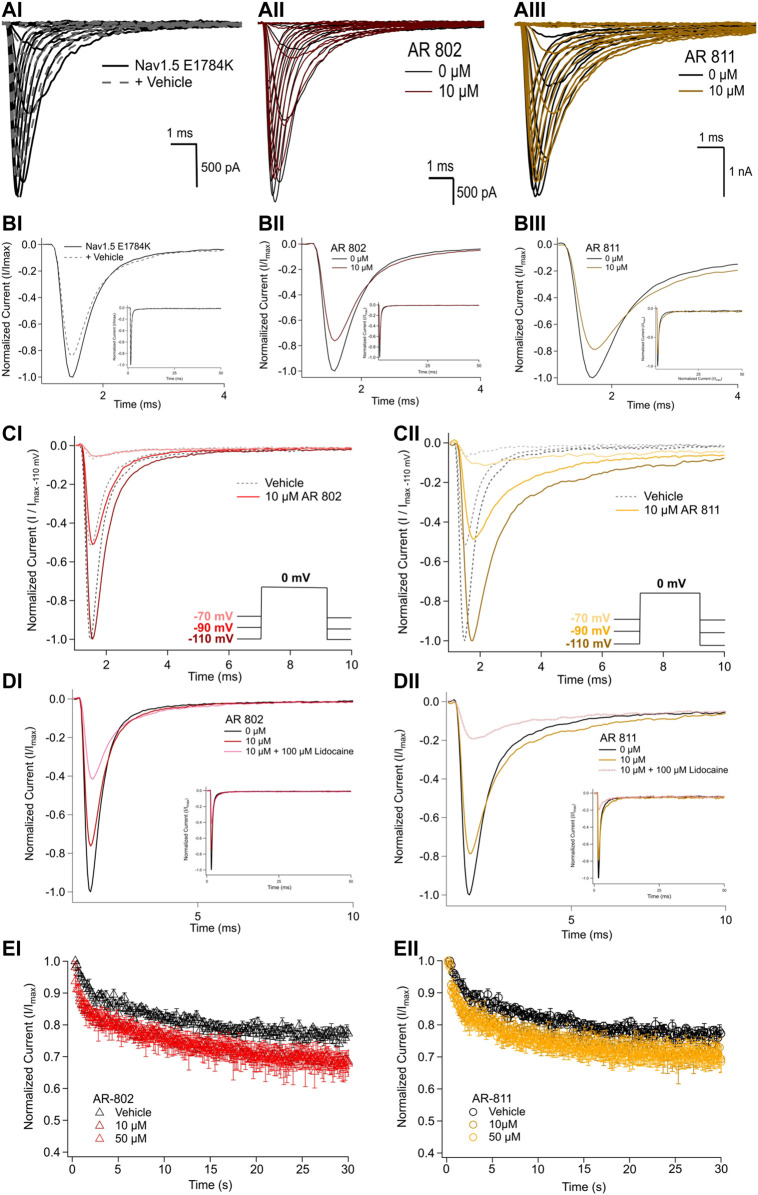
Effects of AR-802 and AR-811 on E1784K. **(AI-III)** show representative traces for peak I_Na_ in vehicle (black) and compound treatment (varied colors, 10 µM) in E1784K. **(BI-III)** show representative traces emphasizing the deceleration in fast inactivation onset kinetics caused by AR-802 and AR-811 compared to vehicle. AR-802 and AR-811 had no significant effect on peak and persistent I_Na_ as shown in the insets. **(CI-CII)** show the deceleration effect of AR-802 and AR-811 on fast inactivation onset when peak I_Na_ is measured after pre-conditioning channels at −110 mV, −90 mV, and −70 mV for 1 s as described in the *Methods*. **(DI-DII)** show the effects of both compounds with the application of 100 µM lidocaine. **(EI-EII)** show the effects of AR-802 and AR-811 on use-dependence.

To confirm their effects in potentiating I_Na_, we screened representative *ARumenamide* compounds, namely AR-802 and AR-811, on the Brugada Syndrome Type 1 and Long-QT syndrome Type 3 mutant, E1784K. Both compounds had no significant effect on peak I_Na_ measured at −20 mV compared to the vehicle control (Figure 6AI-BIII and Table 7). Neither compound affected the midpoint potential value or the slope of conductance (Table 7). Fast inactivation onset kinetics were assessed at voltages greater than −50 mV recorded with the current-voltage protocol. AR-802 and AR-811 had no significant shifts on fast inactivation onset kinetics compared to vehicle (Table 8). However, AR-802 and AR-811 significantly decelerated fast inactivation onset kinetics compared to vehicle in peak I_Na_ measured at 0 mV after E1784K channels were pre-conditioned to −110 mV, −90 mV, and 70 mV for 1 s (Figure 6CI-CII). AR-811 seems to have a larger effect in decelerating fast inactivation kinetics compared to AR-802. AR-802 and AR-811 had no significant effect on peak and persistent I_Na_ (Figure 6BI-BIII *insets*).

**TABLE 7:** E1784K peak I_NA_ and conductance.

	Normalized Post/Pre-Treatment peak I_Na_ at −20 mV	N	Pre-treatment		Post-treatment		N
			GV-V_1/2_ (mV)	GV-z	GV-V_1/2_ (mV)	GV-z		
**Vehicle (0.05% DMSO)**	0.86 ± 0.03	6	−37.3 ± 2.71	3.41 ± 0.30	−38.5 ± 2.15	3.19 ± 0.25	5
**AR-802 (10 µM)**	0.85 ± 0.05	6	−37.5 ± 2.93	3.61 ± 0.38	−38.8 ± 2.10	3.13 ± 0.22	6
**AR-802 (50 µM)**	0.87 ± 0.06	6	−35.3 ± 3.52	3.68 ± 0.46	−37.2 ± 2.87	3.19 ± 0.27	6
**AR-811 (10 µM)**	0.87 ± 0.03	6	−34.8 ± 2.23	2.99 ± 0.23	−37.6 ± 2.27	2.96 ± 0.25	6
**AR-811 (50 µM)**	0.83 ± 0.03	5	−39.5 ± 3.31	3.84 ± 0.54	−41.0 ± 3.42	3.34 ± 0.40	4

**TABLE 8:** E1784K FI ONSET from I-V relationship.

	Difference post—Pre treatment (∆)			N
	FI -30 mV (ms)	FI -10 mV (ms)	FI +10 mV (ms)		
**Vehicle (0.05% DMSO)**	0.20 ± 0.06	0.17 ± 0.05	0.14 ± 0.04	6
**AR-802 (10 µM)**	0.16 ± 0.07	0.15 ± 0.08	0.15 ± 0.08	6
**AR-802 (50 µM)**	0.16 ± 0.10	0.19 ± 0.04	0.20 ± 0.02	6
**AR-811 (10 µM)**	0.13 ± 0.09	0.19 ± 0.06	0.16 ± 0.06	6
**AR-811 (50 µM)**	0.06 ± 0.05	0.13 ± 0.03	0.07 ± 0.02	5

The difference between the post and pre-treatment fast inactivation time constant was significantly larger in AR-802 and AR-811 compared to vehicle when measured from a pre-conditioning pulse of −110 mV (Table 9 and Figure 6CI-CII). To confirm this result, we co-administered lidocaine (100 µM) with AR-802 and AR-811. Lidocaine blocked peak I_Na_ by ∼60%; however, the *AR* effects on fast inactivation kinetics persisted even in the presence of lidocaine (Figure 6DI-DII). Although not shown, washout of lidocaine reverts the blockade in peak I_Na_. Both AR-802 and AR-811 had no effect on use-dependence in E1784K.

**TABLE 9:** E1784K FI onset from persistent I_NA_ (−110 mV).

Difference post—Pre treatment (∆)		N
**Vehicle (0.05% DMSO)**	0.11 ± 0.05	5
**AR-802 (10 µM)**	0.16 ± 0.05	5
**AR-802 (50 µM)**	0.38 ± 0.04*^1^, *^2^	6
**AR-811 (10 µM)**	0.37 ± 0.05*^1^	5
**AR-811 (50 µM)**	0.31 ± 0.05	5

*^1^ *p* < 0.05 vs. Vehicle.

*^2^ *p* < 0.05 vs. 10 µM of AR-802.

## Discussion

Here we introduce a novel class of compounds with the potential to correct LoF in Nav1.5 and arrhythmias that result from LoF. Most available antiarrhythmics that target Nav1.5 block the central pore (peak) I_Na_ to attenuate channel activity and thereby suppress GoF arrhythmias (Antzelevitch et al., 2007; Holmes et al., 2009; Antzelevitch, 2013; Potet et al., 2015). Most GoF conditions in Nav1.5 result in Long-QT syndromes that often degenerate into more lethal forms of arrhythmias, including torsade-de-pointes or ventricular tachycardia and ventricular fibrillation (Gimrikh et al., 1985; Antzelevitch et al., 1996). The underlying pathophysiology is an instability in channel inactivation; therefore, Nav1.5 becomes active during the refractory period of the cardiac action potential and may trigger early or delayed depolarizations (Ten Tusscher et al., 2004; Flaim et al., 2006; Burashnikov et al., 2008). Suppression of I_Na_ is a potent approach for treating these forms of arrhythmias.

Other cardiac conditions, including progressive cardiac conduction disorder (PCCD), sick sinus syndrome, progressive familial heart block, atrial fibrillation, sudden infant death syndrome, and dilated cardiomyopathy, also degenerate into lethal arrhythmias (Savio-Galimberti et al., 2017; Baruteau et al., 2018; Villarreal-Molina et al., 2021). However, they may arise from LoF in Nav1.5 (Amin et al., 2009; 2010b; 2010a). The pathophysiology involves a variety of structural rearrangements in the voltage sensors, pore-forming or voltage-sensing domains, central pore, or the fenestrations (O’Reilly et al., 2012; Payandeh et al., 2012). The effects of LoF on the cardiac action potential are not traditionally treated with Nav1.5 activators but with transient outward potassium current suppressors like quinidine to counteract the repolarizing reserve in light of the suppressed depolarizing current (Brugada et al., 2000; Antzelevitch and Fish, 2006; Wu et al., 2008; Brodie et al., 2018). Toxins like batrachotoxin do have the potential to become Nav1.5 potentiators (Du et al., 2011; Finol-Urdaneta et al., 2019; MacKenzie et al., 2022). However, their harmful effects outweigh their potential benefits.

Although our approach in treating LoF in Nav1.5 is novel, it builds on Hille’s theory (1977) that lipophilic drugs access their binding sites via an alternative route, slowly slithering through the fenestrations into the central pore (Hille, 1977). Structural studies have proven the existence of these fenestrations that Hille correctly predicted (Payandeh et al., 2011, 2012; Kaczmarski and Corry, 2014). The *ARumenamide* compounds we screened were selected from a cohort of other hits based on their affinity for the fenestrations. Although docking *ARumenamides* against the Nav1.5 model based on the NavAb template is temperamental, this approach allowed us to shortlist compounds for electrophysiological testing. We tested our hypothesis that *ARumenamides* block the fenestrations and restrict the motion of Nav1.5 from naturally progressing into inactivation, thereby maintaining I_Na_ conduction. Although we are the first to treat LoF by targeting the fenestrations, previous attempts have been made to attenuate hyperexcitability in Nav through “non-blocking” modulation as with riluzole, which indirectly blocks peak I_Na_ in Nav1.4 by binding to one of the fenestrations (Földi et al., 2021). Our results suggest that AR-802 and AR-811 may be efficacious in attenuating LoF in E1784K. Future structural modelling using molecular dynamic simulations may help confirm our predictions of how *ARs* interact with Nav1.5.

### Interpretation of results

Our results suggest AR-674 differs from the other *AR* candidates in its effects on Nav1.5. Although virtual docking suggested its high binding affinity for the fenestrations, AR-674 blocks I_Na_ and suppresses Nav1.5 availability with time via use-dependence. Independent of any particular theory, the biophysical shifts differentiating AR-674 from the other compounds, or at least its counter sulfonamides that share the same backbone structure, seems to be that its functional group is aliphatic and not aromatic. We predict that an inverse relationship exists between *AR’s* aromaticity and I_Na_ block. The aliphaticity of AR-674 appears to lower the affinity to bind within the fenestrations. Lower affinity within the fenestrations would be predicted to allow greater access of AR-674 into the central pore, which may help explain the 34% reduction in peak I_Na_ at -20 mV and the significant use-dependence at concentrations as low as 10 µM. AR-674 may also interact with the voltage sensors during activation, suppressing their movement and accounting for the decreased slope in conductance.

Aromatic functional groups in *ARs* seem to increase binding affinity within Nav1.5 fenestrations. AR-802, AR-807, and AR-811 have aromatic benzene in their side chains compared to the aliphatic side chain of AR-674. Compound binding within the fenestrations may impede changes in fenestration size during fast inactivation. These three compounds slow the onset of fast inactivation at different concentrations. The fluorine atom attached to the benzene ring of AR-811 seems to mediate the potentiator effects in Nav1.5; however, this conclusion is based only on comparing AR-811 effects with other relatable sulfonamides. The presence of the fluorine atom may account for the high resistance to peak I_Na_ block and the unaltered use-dependence. These results suggest that AR-811 mediates its apparent “potentiating” effects (compared to AR-674) at elevated depolarization rates through an open/inactivated compound-block mechanism. The observed deceleration in fast inactivation kinetics caused by AR-802 and AR-811 is seen only after E1784K channels are pre-conditioned at −110 mV, −90 mV, and −70 mV for 1 s. With prolonged depolarizing pre-conditioning pulses, it seems reasonable to hypothesize that AR-811 mediates its “potentiating” effects by targeting fast and potentially slow inactivation. The benefit of attenuating LoF in E1784K via the aromatic compounds, AR-802 and AR-811, in particular, is that their speculated adherence to the fenestrations reduces their likelihood of blocking peak or persistent I_Na_, a result that we clearly observed.

Compared to the carboxamides, the sulfonamides, AR-051 and AR-189, also inform us about the structural features required to generate Nav1.5 potentiators. Although not statistically significant, AR-189 seems to be slightly better in not suppressing I_Na_ as much at 10 µM compared to AR-051. The branched alkyl side chain adjacent to the furan may increase the hydrophobicity of AR-189, increasing its binding affinity within the fenestrations.

### Study limitations

Virtual docking and the Nav1.5 homology models have limitations. Not all the fenestrations were targetable, especially in rNav1.5. Crystallography and recent cryo-EM structures do not fully depict the fenestrations in their various voltage-dependent conformations (Payandeh et al., 2011, 2012; Shen et al., 2017; Yan et al., 2017; Jiang et al., 2020). Thus, our homology models and the auto-docking experiments were limited by the constraints of these structures. Due to the existent varieties in structural data, it is difficult to determine the exact state dependence of fenestration size. We cannot completely conclude whether the *AR* compounds have preference for a particular fenestration based on the virtual docking alone; however, our modeling clearly showed an affinity of AR compounds screened to the Domain III-IV fenestration in Nav1.5-NavAb and Domain VI-I fenestration in rNav1.5. Molecular dynamics simulation could be used to verify *AR* interaction with the fenestrations in Nav1.5. Additional screening of *AR* compounds against other Brugada variants of Nav1.5 will help confirm our initial experiments that suggest *ARs* may have therapeutic potential to treat LoF.

Treating LoF in Nav1.5 is difficult, given that most compounds will occlude the pore at a certain concentration. Despite the recent advances in designing pharmaceuticals that preferentially inhibit persistent I_Na_ and attenuate GoF, drugs like GS-967, eleclazine, ranolazine, and mexiletine analogs block peak I_Na_ at varying concentrations (Potet et al., 2020; Johnson et al., 2021). To overcome this challenge, we plan to optimize structures like AR-811 that block peak I_Na_ less and decelerate fast inactivation onset. Enhancing the aromaticity of the *ARumenamides’* functional groups is key to enhancing their anti-LoF effects.

### Significance

Our results suggest that *ARumenamides* may have therapeutic potential to treat LoF channelopathies like Brugada Syndrome by lodging within the fenestrations. Although other binding sites are possible, our docking results suggest that these compounds preferentially bind to the fenestrations. *ARs* with aromatic functional groups, as opposed to aliphatic ones, maintained Nav1.5 availability since the bulkier aromatic side groups have a higher affinity for binding within the hydrophobic milieu of the fenestrations. These compounds seem to remain within the fenestrations rather than slithering into the central pore to occlude I_Na_, as opposed to most therapeutic agents targeting Nav1.5. AR-802 and AR-811 (two representative compounds that preferentially delay fast inactivation) attenuate LoF in the most common Brugada Syndrome Type 1 and Long-QT Syndrome Type 3 mutant, E1784K, by decelerating fast inactivation onset kinetics. Neither AR-802 nor AR-811 block peak I_Na_ or enhance persistent I_Na_ in E1784K, two highly sensitive parameters that must be controlled to avoid exacerbation of phenotype. Successfully optimizing *AR* compounds and testing them in LoF models related to cardiac disorders may ultimately lead to lowering the economic burden and health costs associated with untreated arrhythmias.

## Data Availability

The original contributions presented in the study are included in the article/Supplementary Material, further inquiries can be directed to the corresponding author.
